# A Qualitative Exploration of the Referral Process of Children with Common Infections from Private Low-Level Health Facilities in Western Uganda

**DOI:** 10.3390/children8110996

**Published:** 2021-11-02

**Authors:** Juliet Mwanga-Amumpaire, Joan Nakayaga Kalyango, Grace Ndeezi, Joseph Rujumba, Judith Owokuhaisa, Cecilia Stålsby Lundborg, Tobias Alfvén, Celestino Obua, Karin Källander

**Affiliations:** 1 Faculty of Medicine, Mbarara University of Science and Technology, Mbarara P.O. Box 1410, Uganda; jowokuhaisa@must.ac.ug (J.O.); celestino.obua@must.ac.ug (C.O.); 2 Clinical Epidemiology Unit, College of Health Sciences, Makerere University, Kampala P.O. Box 7062, Uganda; nakayaga2001@yahoo.com; 3 Department of Pharmacy, College of Health Sciences, Makerere University, Kampala P.O. Box 7062, Uganda; 4 Department of Pediatrics and Child Health, College of Health Sciences, Makerere University, Kampala P.O. Box 7062, Uganda; gndeezi@gmail.com (G.N.); jrujumba@yahoo.com (J.R.); 5 Department of Global Public Health, Karolinska Institutet, 171 77 Stockholm, Sweden; cecilia.stalsby.lundborg@ki.se (C.S.L.); tobias.alfven@ki.se (T.A.); karin.kallander@ki.se (K.K.); 6 Sachs’ Children and Youth Hospital, 118 83 Stockholm, Sweden; 7 Programme Division, Health Section, UNICEF, New York, NY 10017, USA

**Keywords:** referral, primary private health facilities, low-income countries

## Abstract

Over 50% of sick children are treated by private primary-level facilities, but data on patient referral processes from such facilities are limited. We explored the perspectives of healthcare providers and child caretakers on the referral process of children with common childhood infections from private low-level health facilities in Mbarara District. We carried out 43 in-depth interviews with health workers and caretakers of sick children, purposively selected from 30 facilities, until data saturation was achieved. The issues discussed included the process of referral, challenges in referral completion and ways to improve the process. We used thematic analysis, using a combined deductive/inductive approach. The reasons for where and how to refer were shaped by the patients’ clinical characteristics, the caretakers’ ability to pay and health workers’ perceptions. Caretaker non-adherence to referral and inadequate communication between health facilities were the major challenges to the referral process. Suggestions for improving referrals were hinged on procedures to promote caretaker adherence to referral, including reducing waiting time and minimising the expenses incurred by caretakers. We recommend that triage at referral facilities should be improved and that health workers in low-level private health facilities (LLPHFs) should routinely be included in the capacity-building trainings organised by the Ministry of Health (MoH) and in workshops to disseminate health policies and national healthcare guidelines. Further research should be done on the effect of improving communication between LLPHFs and referral health facilities by affordable means, such as telephone, and the impact of community initiatives, such as transport vouchers, on promoting adherence to referral for sick children.

## 1. Introduction

About 2,000,000 deaths of under-5-year-old children still occur every year in sub-Saharan Africa (SSA), mainly from malaria, diarrhoea, pneumonia and neonatal infections [1,2]. In Uganda, some progress has been made and the under-5 mortality now stands at 46.4 deaths per 1000 live births but is still above the SDG 3 target [3,4]. Prompt and appropriate care for children with infectious diseases can contribute to reducing child deaths and to realising the SDG targets. While severe forms of infectious diseases, such as cerebral malaria, are rarer than uncomplicated conditions, the case fatality of the severe forms is higher [5,6]. Over 50% of sick children first receive healthcare from private primary health facilities in Uganda; however, many of these facilities lack the capacity to manage complicated conditions, such as severe pneumonia and severe malaria [7,8]. Such cases should be referred for escalation of care, and therefore a well-streamlined referral system is important. It is important that the healthcare providers have the competence to identify and appropriately refer sick children needing referral.

The World Health Organization (WHO) defines referral as the process in which a health worker at one level of the health system, with insufficient resources to manage a clinical condition, seeks the assistance from a better-resourced facility at the same or higher level [9]. It is part of the processes of care for children in the WHO Integrated Management of Childhood Illnesses (IMCI) strategy [10,11]. When IMCI is carried out appropriately, 16–46% of children will require referral, and in developing countries, 10–20% of sick children are referred to hospitals for further care [12,13,14,15].

At lower levels of healthcare, it is not automatic that sick children are referred promptly and studies have shown that clinical assessment and severity classification of conditions such as pneumonia is challenging [16]. In addition, not all referrals are adhered to; studies from rural Uganda carried out among community health workers and public health facilities show that less than 50% of caregivers of children referred complete the process [17,18,19]. Reasons for noncompliance with referral often include lack of money, long distance to referral health facilities coupled with difficulties in accessing transport, lack of someone to attend to the home and other children, failure by the caretaker to recognise that the illness is serious and not appreciating the importance of referral. Some other challenges result from the units to which the sick children have been referred delaying to recognise the urgent care needed by the sick children, perceived poor quality of care at referral facilities, lack of connection between different levels of the referral systems and insufficient knowledge about the referral system [17,18,19,20]. We explored perspectives of healthcare providers and child caretakers regarding the process and challenges in referral of children with common childhood infections from low-level private health facilities (LLPHFs) in Mbarara District in order to identify and advocate for ways of improving referral from such facilities as a form of promoting equity of care in rural areas.

## 2. Materials and Methods

### 2.1. Research Design and Setting

This qualitative study was nested in a larger quantitative survey of quality of healthcare for common paediatric infections in 110 LLPHFs selected randomly in Mbarara District [21]. The qualitative design was used to enable an in-depth exploration and understanding of healthcare workers’ and caretakers’ own experiences and perspectives regarding referral practices by LLPHFs providing child health services in Mbarara District. Mbarara is located 267 km south-west of Kampala, Uganda, and by the time of implementation of this study included 3 administrative counties, 16 sub-counties, 83 parishes and 742 villages. While the district recently acquired a city status, it is primarily rural. By the time this study was conceived, the district had a population of about 470,000 inhabitants and a density of 99 inhabitants/km. The main health facility is the Mbarara Regional Referral Hospital (MRRH), a tertiary referral centre that also serves as a teaching hospital for the Mbarara University medical school. The health facilities in the district follow the Uganda Ministry of Health (MoH) facility classification defined by the population, catchment area served and services offered [22], as illustrated in Table 1.

The Ugandan healthcare system is pluralistic; care being provided by public and private entities. The private health facilities levels follow the MoH facility classification. Patients from private health facilities can be referred to either other private or public health facilities with capacity for more advanced care.

About 124 private facilities, uniformly distributed throughout the district, were registered with the national regulatory authorities in 2017 when this study was conceived. Of these, six are hospitals, while the majority of the others are at the level of health centre (HC) III or lower, as described in Table 1. For the purposes of this study, we defined such facilities as low-level private health facilities (LLPHFs). These LLPHFs have minimal infrastructure and treat common paediatric diseases, such as uncomplicated malaria, pneumonia and diarrhoea, and may provide immunisation. A few have small in-patient services and a laboratory for simple diagnostics, such as malaria blood slides and urine tests. Many private clinics are run by nurses or midwives and clinical officers whose highest level of qualification is certificates or diplomas, while a few are headed by medical doctors with medical degrees. In more rural facilities, the services may even be delivered by individuals with minimal health-related training.

### 2.2. Sampling and Recruitment

**Figure 1:**
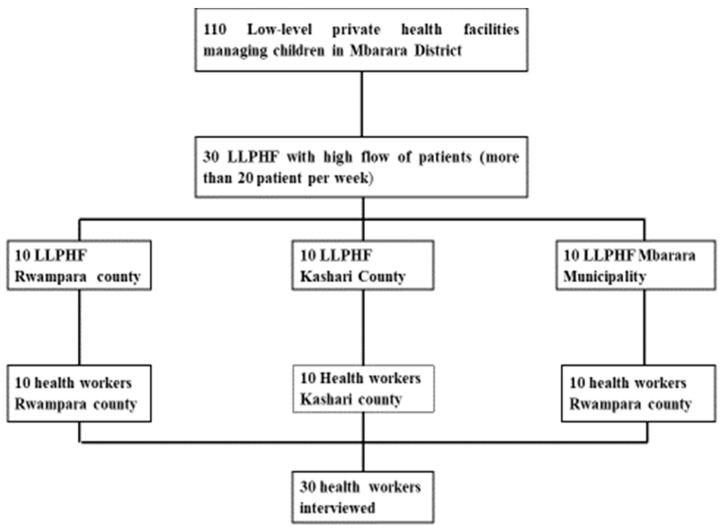
Flowchart for purposive sampling of health facility and healthcare worker participants for perspectives of HCW on the referral process from low-level health facilities in Mbarara District.

Key informants with rich experience pertinent to the study topic were selected using purposive sampling. They included healthcare providers from 30 purposively selected LLPHFs that offered child health services, received at least 20 patients per week and had been in operation for at least 2 years, as shown in Figure 1. The healthcare providers were identified with the help of the facility heads and were included if:

1. They were used at an LLPHF; and
2. Had been providing clinical care for children in a private facility for at least 6 months.

They included nursing assistants, nurses/midwives, clinical officers and medical doctors. Caretakers were included if their child had been referred for further care. Ten health facilities were picked from each of the three counties, and only one healthcare provider was interviewed from each of these LLPHFs. This enabled capturing the heterogeneity among the healthcare providers and health facilities and allowed for inclusion from rural and urban settings. We also purposively sampled caretakers of severely ill children who had been referred for further care from these LLPHFs. Caretakers of children who had been referred were approached while at the LLPHF and asked whether they would accept to be interviewed at a later date. If they accepted, their phone contacts and addresses were taken. Three to five days after the referral date, they were called and asked whether they were still willing to participate in the interview. This allowed time for the child to stabilise or recover so that the caretaker could be engaged comfortably in the interview. If they accepted, the research assistant (RA) then went either to their home or the hospital if the child was still admitted. Only 16 out of the 21 caretakers who had given their contacts were interviewed because there was no more new information coming out of additional interviews.

All individuals who were approached agreed to participate in the study. The sample size of 30 health provider participants and 16 caretakers was determined through data saturation or the point when additional interviews did not provide novel insight or point to new concepts [23,24]. All participants provided written informed consent, including permission to audio-record the interviews. After completion of the interviews, participants received 20,000 Uganda shillings (~$5 US dollars) as compensation for participation, the standard for research studies in the country.

### 2.3. Data Collection

In-depth interviews were conducted between May and December 2019 by two female Ugandan research assistants (RAs) with prior training and experience in qualitative research. Both interviewers were fluent in English and Runyankore, the dialect spoken by the majority of people in Mbarara. The RAs did not know any of the study participants before conducting the interviews. Prior to study initiation, the RAs were trained for 3 days on the study protocol, principles of qualitative data collection and how to conduct high-quality interviews, interview translation and transcription. An interview guide was specifically created for this study to ensure consistent focus on the topic of the process of referral of sick children from LLPHFs to higher levels of care, the challenges faced in referral and how to improve these processes among participants. The interview guide questions were developed using input from Mbarara District Health Team (DHT) officials. The DHT is the technical health sector decision-making and planning body at the district level and is headed by the district health officer. The interview guide was piloted at 3 private facilities, while that for caretakers was piloted on 3 caretakers of children admitted to the paediatric ward of the Mbarara Regional Hospital, but the responses were not included in the analysis. The interviews were conducted in a private room at the respondents’ respective workplaces for the healthcare providers. For the caretakers, the interviews took place at their homes (*n* = 6) or at the hospital to which they had been referred (*n* = 10). One mother whose child passed on was interviewed 3 months after the event to allow for mourning. Interviews took place at a time and in a language preferred by the participants. All the 30 provider interviews were carried out in the English language, while all the 16 caretaker interviews were carried out in Runyankore. Each interview lasted for approximately 60 min and was audio-recorded with participant’s permission. All interviews were transcribed verbatim by the interviewer based on audio recordings. The 16 interviews conducted in Runyankore were concurrently translated into English and transcribed. All transcripts were proofread by the first author, who speaks both languages fluently, for quality and translational integrity. The first author read the transcripts line by line within 72 h of transcript completion and provided feedback to the RAs to continuously improve their interview skills throughout the data collection period. This ensured consistency in quality and content across all interviews and served as a means to monitor for data saturation.

### 2.4. Analytical Process

Interview data were analysed using thematic analysis using the combined deductive/inductive approach [25,26,27]. Two of the authors reviewed each transcripts several times and independently developed an initial set of codes. The two authors then discussed and compared their codebooks. Through consensus, the codes were revised to create a final code list, which was grouped together to identify subthemes and themes pertinent to the research question. A preliminary list of subthemes and themes were shared and discussed with the authors and one independent peer who is a paediatrician with experience in qualitative research. Through further discussion, final themes and corresponding subthemes in relation to the referral of sick children from LLPHFs were developed and are presented here with illustrative quotes from interview transcripts. The research team was made up of healthcare workers, researchers and academicians with different expertise, nationalities and backgrounds. We reflected on the impact of our backgrounds on the different phases of the research process. ATLAS.ti Scientific Software Development GmbH (GmbH, Berlin) was used for data organisation [28].

### 2.5. Ethical Approval

The study was approved by the Makerere University, School of Medicine Research and Ethics Committee (SOM-REC; reference no. REC REF 2017-059) on the 15 January 2019 and the Uganda National Council for Science and Technology (UNCST; reference no. SS 4903) on the 23 April 2019.

## 3. Results

### 3.1. Demographics Characteristics of Study Participants

About three-fourths of the caretaker participants were female, nine of whom were mothers to the sick children. The average age of the caretaker participants was 30.8 years. The health worker participants had, on average, been in service for 6.5 years, and most were nurses. The other characteristics of the participants are shown in Table 2.

### 3.2. Overarching Themes

We formulated and identified six themes: reasons for referral, process of referral, health worker attitudes to referral, challenges in referral, experiences of caretakers and how the referral process could be improved. These, along with the subthemes and odes, are illustrated in Table 3.

#### 3.2.1. Reasons for Referral

All participants stated that sick children are referred because they have a severe condition and need advanced care that the LLPHF lacks the capacity to handle. Such incapacities include the health workers having limited knowledge of the disease condition, limited investigative capacity, lack of medical materials such as oxygen and lack of facilities to admit patients for prolonged admission.

*“In most cases, like in neonates, you find that the child has difficulty breathing, and we don’t have an oxygen cylinder or concentrator…. Another reason is that the child may have HIV, in addition to malaria or pneumonia, and is in critical condition, and you need to refer because the child needs additional support and treatment of HIV.”* (HW1, male nurse, Mbarara Municipality)

*“The health worker did not know the exact diagnosis for the child and did not have the treatment as well. She checked for malaria, which was negative, and told me that she did not have any other medication apart from Coartem* (malaria medicine) *and cough medicines. She told me to take the child to experts.”* (CT1, mother of a child with a fever)

Further, health workers often referred children to avoid loss of income if they perceived the caretaker as not having money to pay the services to be provided.

*“If it is [a] simple condition that we can manage but the caretaker cannot afford the payment, then I refer such patients.”* (HW2, male clinical officer, Mbarara Municipality)

About a fifth of HWs at LLPHFs mentioned promptly referring children who were severely ill to avoid the death of the children occurring at their health facilities, which would negatively affect the image of the health facilities.

*“Sometimes we provide the services and even give transport to severely ill patients because you do not want the child to die from your clinic.”* (HW3, female nursing assistant, Rwampara)

#### 3.2.2. Process of Referral: Person Responsible, Where to Refer and How Are Referrals Done

**Person responsible for referring patients**

The person responsible for referral varied for different facilities; however, often it was the health worker who assessed the child, in consultation with the most senior health worker or the proprietor of the facility. This senior person could be a nurse, a doctor or a clinical officer. Sometimes, the owner was even a laboratory technician.

*“Each and every patient who comes, I have to let the doctor know about the condition wherever he is. I don’t tamper with them; he tells me every step to take, how the prognosis is and then comes to review them if possible, then refers them.”* (HW4, female nurse, Kashari)

*“The owner of the clinic determines the referrals. When I get complicated cases, I consult her first, so she is the decision maker.”* (HW5, female nurse, Rwampara)

**Where LLPHFs refer their clients**

Most participants referred the children to facilities with more qualified health providers than themselves or specialised services regardless of whether these facilities were public or private entities. This was either the Mbarara Regional Referral Hospital (MRRH), a public facility, or the Holy Innocents’ Children’s hospital (HICH), a private, not-for-profit entity. Between those two choices, the decision of where to refer was often shaped by the perception of how fast the children would receive care, provision of better medical care and the possibility to receive feedback about the cases referred. Sometimes, the perceptions about the referral facilities differed from one participant to another.

*“I prefer referring to Mbarara Regional Referral Hospital (MRRH) because when they receive a referral, they attend to it immediately….”* (HW6, female nurse, Rwampara)

*“Mbarara is a regional referral facility and has many patients and is overcrowded. When you refer the patient there, they may not go. It’s not that they are not delivering services, but the number of health workers is very small compared to the patients.”* (HW1, male nurse, Mbarara Municipality)

A few HWs referred to other private clinics manned by a person more qualified than themselves, as illustrated by one doctor.

*“As a medical officer without specialisation, sometimes I refer to a specialist physician in another private clinic. I look at it at that level, and there you get good feedback; you learn from the case, and the patient gets helped. But if you send someone in the referral hospital (MRRH), immediately they attend to the patient, but you never get feedback.”* (HW7, male doctor, Mbarara Municipality)

**How referrals from LLPHFs are done**

The format of referrals often depended on the facility, how critically ill the child presented and how long the patient had been treated at the clinic for the particular illness. Participants reported use of referral letters or notes with minimum information, asking the patient to go with the medical forms on which other notes had been written, or verbal referrals with no written document.

*“We usually write referral notes and the treatment if the patient has been here, but in most cases, if we have not touched the patient and have not given any medication yet, we tell them verbally to go. If he has been our client, we write everything we have done and refer.”* (HW1, male nurse, Mbarara Municipality)

In some rare cases, especially where the proprietor was not a medical personnel or worked in a referral hospital, the patients were brought and physically handed over to health workers at the referral facilities.

*“They bring you physically with the health worker, who leaves after handing you over, and they have initiated treatment.”* (CT2, 32-year-old mother of a newborn infant)

#### 3.2.3. Attitudes of the Health Workers about Referring Patients

Most HW participants perceived referring patients out of their health facilities positively because it resulted in good outcomes for the patients, thus increasing trust from their clientele. In addition, it provided an opportunity for learning for some health workers on how to handle similar cases in the future.

*“If you make a call and they say the child is improving, of course you feel good; you say my referral is of advantage now.”* (HW8, female nurse, Mbarara Municipality).

*“Referring cases I cannot handle creates for me confidence and trust from the public because I direct them where they can get proper management. When I get the chance and see the treatment that the child was given or the diagnosis that was made, I learn from it, and next time if I get a similar condition, I treat according to what I have observed.”* (HW9, male nurse, Mbarara Municipality).

However, about a fifth of HW participants were negative about referring patients, as they were anxious about disappointing their clients and feared being perceived as incompetent.

*“You don’t feel good to refer, because it’s a private clinic, and when you refer, they may think you are unable to deliver services very well. People outside may think that you are incompetent.”* (HW4, female nurse, Rwampara).

#### 3.2.4. Challenges to the Referral Process

The most frequent challenges experienced by the HW participants were categorised as:

1. Non-adherence to referral instructions by the caretakers
2. Loss of revenue to the clinic
3. Lack of feedback concerning the referral from referral facilities

**Non-adherence to the referral**

Healthcare worker participants were often challenged by caretakers not adhering to the referral instructions either by refusing to go the facility chosen by the HW or by delaying to depart or failing to go completely. This occurrence was also confirmed by caretaker participants. Lack of funds to cater for transport, upkeep while at referral facilities or payment of medical bills was one of the major reasons mentioned for non-adherence to referral instructions. Caretakers often opted for facilities that they considered more accessible physically.

*“The main problem is most of the caretakers are unable to find money for transport fare when I refer them to the Mbarara or Itojo hospital; they instead go to another private facility around…”* (HW10, female nurse, Rwampara)

*“The health worker referred us to Mbarara (hospital), but we instead went to Kobi, a health facility in our neighbourhood and easy for us to access… we were told the child had no blood, and he died from there.”* (CT3, 29-year-old mother who did not comply with the referral)

Some caretakers complied with the referral but left the referral facilities before discharge due to the inability to afford costs of care. One of the caretakers explained:

*“At the children’s hospital where the doctor had referred us, they cared for the patients well, but we left that hospital because the medical bills were high and we did not have enough money.”* (CT4, 26-year-old father of child with fever and convulsions)

Improvement in the child’s condition was often another reason for not proceeding with the referral.

*“They told me to go to the MRRH, that big hospital; I did not go there, because the child improved when we got the treatment… so since I saw he (child) was ok (improved), I decided not to bother myself.”* (CT5, 23-year-old mother of a child with pneumonia)

**Loss of revenue to the clinic**

Some health workers expressed difficulties in recovering costs on care and referral when the caretaker fails or refuses to pay for the pre-referral services. This often prevents clinics from giving pre-referral treatment to patients.

*“When we refer them, they feel like we have done nothing for their children. They resist paying us for the care received before referral, because they believe we have failed.”* (HW11, male nurse, Mbarara Municipality)

**Lack of communication and feedback on the final outcome of the patients’ condition**

HWs expressed frustration at the communication gap between LLPHFs and higher health facilities, especially public hospitals. Most LLPHF health workers mentioned they often never get to know the outcome of the referrals they make, because of a lack of feedback from the higher facilities where the children have been referred.

*“They* (the referral health facility) *are supposed to fill what was done on the form at the time of discharge so that if the patient comes back, you get to know what was done. But we never get any feedback….”* (HW7, male doctor, Mbarara Municipality)

#### 3.2.5. Experiences of Caregivers during the Referral Process

Caregivers expressed negative and positive experiences during the referral process, sometimes contradicting each other. Negative experiences included:

1. Incurring high costs for transport, medical care and feeding
2. Difficulty in accessing transport
3. Overcrowding on the ward spaces
4. Unfriendly health facility staff
5. Delays in accessing care

Positive experiences included the following:

1. Possibility for the child to receive appropriate care
2. Caring health workers
3. Not having to pay for medical care

**Incurring high costs and difficult transport**

Participants reported that they had incurred high costs while at the referral facilities, and sometimes, they did not have the money to even take care of basic needs, such as buying food.

*“Here, the expenditures are high because I had to pay for the scan and blood investigations. I no longer have any money to even buy food.”* (CT6, 45-year-old grandmother of a newborn, at a public hospital)

*“I could not afford to buy a full dose of treatment for the baby, because I came expecting free services and now I have to pay….”* (CT7, father of a 6-month-old with pneumonia, at a public hospital)

Participants also experienced difficulties with transportation, and often, the only means of transport available was motorcycles, which were perceived as an unsafe mode of transport with increased risk of road traffic accidents and exposing the sick children to the cold wind. It was often impossible to use cars or ambulances because the caregiver did not have money to pay for the hire or because they resided in places with poor roads.

*“They told me that the ambulance would cost UGX 70,000* (USD 19), *and I only had UGX 10,000, so I took a bodaboda* (taxi motorbike) *that left me on the main road….”* (CT8, 50-year-old grandmother of a newborn)

*“…. the roads from our place have not been worked on; they have stones, so reaching here with the patient is a hard process.”* (CT6, 45-year-old grandmother of a newborn)

*“The clinic doctor said the child was badly off because the cold wind affected her, and she got pneumonia. Now we had to again take the same child on a bodaboda. She was going to get worse….”* (CT9, 25-year-old father of a child with pneumonia)

**Overcrowding on ward spaces**

The participants expressed their discomfort with the congestion they experienced on the wards, especially in public hospitals.

*“They work from very small rooms congested with patients and machines. There is even no space for the one who is weighing the babies. You just peep through the glass to see if the doctor has finished seeing the baby.”* (CT10, 25-year-old mother of a child with pneumonia, in a public hospital)

**Unfriendly facility staff**

Participants reported to have encountered some health facility staff at the public referral sites, including health workers and support staff, who did not handle them well.

*“The medical personnel need to learn how to speak properly to the patients… I am already shuttered with [my] child’s sickness, so if you just shout at me, I don’t feel good.”* (CT10, 25-year-old mother of a child with pneumonia)

*“What annoyed us are the cleaning staff at the hospital, who abused us and poured water on us while we were sleeping….”* (CT6, 45-year-old grandmother in a public hospital)

**Delays in receiving medical care**

Participants reported delays at various points in receiving care, especially at public referral facilities. They waited long before the child was assessed, and they waited long at the laboratory for specimen to be taken off and sometimes even to receive their discharge documents.

*“I did not find a conducive situation for receiving very sick children at the main referral hospital. My husband wasn’t happy with the way they were not minding about us, and as a parent, you panic thinking that the child might die, so he decided to go to Holy Innocents* (a private children’s hospital)…” (CT10, 25-year-old mother of a child with pneumonia)

*“We spent a long time at the laboratory. I reached there at 9:00 am or 9:30 am and left at 12 midday. The laboratory personnel were not busy but were concentrating on their phones…”* (CT11, mother of a child with fever)

In contrast, some caretaker participants acknowledged good experiences from the referral facilities. These included prompt and good medical care; positive experiences were more likely to be reported when there was good communication and explanations from the health workers.

*“At the other clinic, we had spent 3 days, but the child’s condition was worsening…but when we reached the [Mbarara] Regional Referral Hospital, they quickly helped us; they put her on oxygen, checked her blood to confirm the disease and later gave her treatment through the cannula which they put on the hand.”* (CT12, 36-year-old father of a child with pneumonia)

*“At the regional hospital, the health workers cared for my baby a lot. As a parent, I could not understand what the health workers were doing, but you could see them moving here and there, doing this and that, explaining to me why they are doing somethings, assuring me that the baby will be fine. I could see the baby cry, and I thought she had pain when they put the feeding tube, but they explained to me that in her condition, she should not be on empty stomach. They were giving us psychological care and making sure we understood what was going on.”* (CT7, father of a 6-month-old baby with pneumonia)

#### 3.2.6. Views on How to Improve the Referral Process from LLPHFs

The participants’ views regarding how to improve the referral process from LLPHs were shaped by desire to:

1. Reduce the waiting time
2. Improve transportation
3. Reduce costs incurred during the referral process
4. Improve communication

These could be achieved by increasing the health workforce for the hospitals, giving referral letters/notes to patients, addressing transport issues and setting up more hospitals in communities.

**Increase in the health workforce**

Increasing the number of health workers both in LLPHFs and in higher-level hospitals was often mentioned by participants. This would make the referrals smoother, reduce the patient waiting time and reduce staff exhaustion.

*“The government needs to add more doctors because what I saw there was too much; there were very many sick children and only two doctors. This is the reason why we were not worked on quickly. At least, they should be four doctors.”* (CT5, 25-year-old mother of a child with pneumonia, at a public hospital)

*“Private clinics should employ enough staff so that the referred child is accompanied by a nurse regardless of whether there is an ambulance or not and handed over to staff at the referral facility.”* (HW12, male clinical officer, Kashari)

**Referral letter/note**

Participants mentioned the need for referring clinics to give them referral letters as oftentimes the sick children were referred verbally without any referral document. Participants explained that if one was referred with a document, it would be easier for the person receiving them at the referral site to know where to start.

*“Another thing that should be improved is the way we are referred…usually in the clinics, they perform the first tests and so know how the child is. I may not know how to explain this result when I reach the hospital, so it is better if it is written down….”* (CT9, 25-year-old father of a child with pneumonia)

**Improve on transport**

Participants mentioned provision of community ambulances to transport sick children as a means to improve the referral process.

*“Even though some things are not available, they (*government*) need to get for us ambulances….”* (CT12, 36-year-old father)

*“If those bigger facilities could put in place ambulances at a cost that is affordable and even collaborate with the health facilities around…. when you get a condition to refer here, then we contact them.”* (HW10, female nurse, Rwampara)

**Better roads**

Participants also mentioned improvement in the road network, since most come from rural areas with bad roads.

*“The government should make better roads, and where we are referring them for better services”* (HW13, female nurse, Kashari)

**More hospitals nearer to the communities with sufficient supplies**

In addition to stocking the existing public health facilities with sufficient supplies, participants suggested establishing more health facilities in communities so that patients do not have to travel long distances. This would reduce the costs of living and travel.

*“The government should put for us facilities in the village like the one you get from here (MRRH) so that when the children are sick, you go to the nearest hospital and we stop struggling when our children fall sick.”* (CT6, 45-year-old grandmother)

*“They should make sure that in case a child comes and needs oxygen, it should be available. They may not be so many, but the available ones must be in good working condition because when I was still in the hospital, there are some children who died; you could see the health worker struggling, but the oxygen failed, yet the health worker did all she could to save this child’s life.”* (CT7, father of a6 month-old with pneumonia)

**Open communication as a means to improve referrals**

Good communication was identified as a tool for improving the referral process by the health workers giving explanations to the caretakers about why and where they are referring the children. In addition, good communication between the HWs at referring facilities and the facilities where the children were referred was identified as a tool to enable feedback, along with its benefits.

*“The best way to handle this is to tell the clients where our capacity stops during the course of assessment, so that when we refer them they understand our limits.”* (HW14, male nurse, Kashari)

*“When you are referring someone, put your contacts because more explanations may be required of you from the facility you refer the child to, and they may want to give you feedback…”* (HW15, male doctor, Mbarara Municipality)

## 4. Discussion

We explored the referral process of children below 5 years attending LLPHFs in Mbarara District. We found that the reasons to refer, where to refer and how to refer were shaped by the patients’ clinical characteristics, the caretakers’ ability to pay and health workers’ perceptions. Non-adherence to referral by the caretakers and inadequate communication between LLPHFs and higher referral facilities were the major challenges faced by LLPHFs in the referral process. Suggestions for improving the referral process and minimising non-adherence to referral were hinged on procedures that would improve transport of patients and their caretakers to referral sites, reduce waiting time in hospitals and minimise the expenses incurred by caretakers.

**Reasons for referring, where to refer and how referrals are done**

The most common reason given by HW participants in LLPHFs for referring sick children was to enable them to receive better healthcare because they had complex clinical conditions than would be handled at the LLPHFs. In addition, HWs also promptly referred children who were severely ill, for fear of death occurring at their health facilities, which would negatively affect the image of the health facilities. This is contrary to the findings of a study in central Uganda where referring patients was associated with perceived loss of prestige by the clinic [29] but similar to another study where children with danger signs were likely to be referred [18]. A study done in Vietnam found that among the children admitted in intensive care units (ICU), children who were referred in, especially from low-level health facilities, were the more critically ill ones [30]. This underscores the need for receiving health facilities to quickly identify and attend to all children referred from lower facilities urgently as they are likely to be severely ill.

Most HWs from LLPHFs referred to hospitals, especially Mbarara Regional Referral Hospital or to the children’s specialised hospital other than facilities below the hospital level. This is comparable to what other studies have described in Uganda [8,18,29], where most referrals from private clinics are sent to public hospitals. Most LLPHFs have minimum capacity with HWs who may have limited knowledge, inadequate infrastructure, lack of equipment and materials for managing critically ill children, such as oxygen, and often limited laboratory services as has been described in Nigeria [31]. To increase financial risk protection of households against impoverishment due to health expenditure, the Ugandan MoH planned on upgrading the status of all public HCII medical facilities to HCIII medical facilities and creating community hospitals [32]. If achieved, this will contribute to achieving universal health coverage (UHC).

In most cases, health workers were positive about referring sick children. However, a few expressed anxiety about being perceived as incompetent by the caretakers if they referred patients. This anxiety is not unfounded, as caretakers in a study carried out in central Uganda perceived health workers who referred sick children as incompetent [29]. While in limited-resource settings, the reasons for failure to refer include logistic encumbrances to caretakers, fear of loss of trust and prestige and fear of financial loss, in developed countries, such as the United States, reduced referrals from primary health physicians is mainly because of increased and improved care facilities in rural areas [29,33,34]. This could be emulated in the Ugandan context and improve the quality of healthcare provided in these LLPHFs. Referral letters were given in only a few cases, but many HCWs referred the patients verbally. This lack of referral documents was also noted previously in a Ugandan study and in an Ethiopian study where less than half of the sick children were given referral forms [8,33]. Referral letters are important as they facilitate a severely ill child to receive care quickly at a higher facility.

**Challenges to the referral process**

Non-adherence to referral was a common challenge mentioned in this study, often as a result of difficulties in transportation for caretakers, lack of funds to meet other costs of the referral, a long waiting time before the child accesses medical care and perceived improvement in the child’s condition after initial treatment. These findings are comparable to results of other studies carried out in Uganda and other African countries. A study describing caretaker compliance to referral by community health workers in Uganda and Tanzania found low compliance when pre-referral treatment was administered, as this led to temporary improvement in symptoms the child had [18,35,36]. Studies done in central and eastern Uganda showed low adherence rates, with only 10% and 28% of caretakers complying to referrals for their sick children from community health workers [17,36]. A study done in rural Nigeria for referrals of infants with possible serious bacterial infections found that over 90% of caretakers did not comply with the referral advice to take the child to hospital [37] and the reasons for noncompliance were dysfunctional referral sites with no drugs and HWs. Transportation difficulties and high maintenance costs while at referral sites are common challenges that have also been described in other settings. Indeed, other studies in Africa, including Uganda, and other parts of the world have identified difficulties of transport and the high cost of sustenance as major challenges as well [18,38,39,40]. The negative experiences that caretakers go through at referral sites, including long waiting times, high costs of living, unfriendly facility staff and transport difficulties, compound the noncompliance to referral. A study carried out to assess access to healthcare for under-5-year-olds in 12 districts in Uganda identified most of these factors as barriers to care seeking [40]. These negative experiences can augment non-adherence in the future and also affect the choice of where to seek care, as has already been described by other researchers [35,39,41,42]. A systematic review in the United States assessing differences in seeking care between urban and rural areas of residence also found that geographic distance, long waiting times and costs are barriers to seeking specialist care for minority communities [34]. On the contrary, in Afghanistan, Newbrander et al. found that transport costs do not lead to non-adherence, probably because the patients do not have to travel long distances to seek care [39]. This underscores the need for making transportation easier for the people or bringing the services closer to them. Another challenge mentioned by the HWs in LLPHFs was inadequate communication between the private facility staff and those in referral facilities. This is an area for more research, as we did not find any studies describing this phenomenon in Uganda; however, it has been described by researchers in the United States and India [43,44]. Lack of communication leads to frustration as the HWs who refer never get to know how the patient is faring. This is also a missed opportunity for the lower-cadre HWs to learn how to manage similar cases in future. In contrast, referring HWs sometimes refer patients with minimal or no referral letters at all. This disadvantages the patient as they have to start afresh when they reach the referral sites, thus increasing the waiting time further. Research has shown that children referred from lower health facilities are more critically ill and have poorer outcomes [30] and that referral notes and explaining the reasons for referral to caretakers facilitate adherence to referral advice [39]. Further, research shows that apart from facilitating adherence to referral advice, well-written referral letters lead to patients presenting in less critical condition [39,44]. Delayed referrals or noncompliance to referral may result in poor outcomes as the children may present in a more critical condition or even die before receiving appropriate care, as was found in previous study in Uganda [17,38].

**Suggestions for improving the referral process**

To improve the referral process, strategies that could reduce the waiting time for patients in hospitals, reduce referral costs incurred by caretakers and improve communication between referring and recipient health facilities are important. Inadequate human resource for health contributes to delays in attending to patients. According to the Annual Health Sector Performance Report (AHSPR) of 2018/2019, the goal of the MoH is to have enough, competent, equitably distributed, motivated and facilitated health workers at all levels of the system. Despite the financial challenges made worse by outbreaks and unplanned catastrophes, there is the goodwill of the government, and efforts are in place by the MoH to raise appropriate staffing levels in public facilities to above the 80% target of the health sector development plan (HSDP) [22,32,45]. In the meantime, it is necessary to set clear referral guidelines, train HCWs at LLPHFs to write proper referral letters and establish communication networks between health facilities of different levels, as has successfully been done in Kenya [46]. These suggestions relate to what the MoH spelt out in the HSDP 2015/2016–2019/2020 but have not been realised. This is attributed to the COVID-19 pandemic and other health system issues that have diverted funding and other efforts [32,45]. It is important for the district health office to work with researchers to advocate for and suggest to local political leaders innovative and cost-effective solutions to improve the referral process at low-level health facilities. These could include community initiatives, such as giving transport vouchers to caretakers whose child has been referred, as was done to improve health facility deliveries in northern Uganda [47]. This would be in keeping with the current Parish model of rural development and the 3rd Uganda National Development Plan, whose goal is to increase the average household income and improve the quality of life for Ugandans [48].

A limitation of this paper is that we did not interview health workers at facilities to which the patients are referred in order to capture their perspective and clarify some of the issues put forward by caretakers and referring facilities. However, the fact that we interviewed caretakers of patients who had been referred shortly after is a strength to this paper because their lived experiences were still fresh in their minds.

## 5. Conclusions

Health workers in LLPHFs have positive attitudes towards referring sick children, but key challenges exist, which lead to caretaker non-adherence to referral. These include health system issues, such as long waiting times, inadequate medicines and materials for patient care and a large number of patients at public referral health facilities compared to the healthcare workers. Caretaker-related challenges include high referral-related costs and transport challenges. We recommend that triage at referral facilities should be improved and that health workers in LLPHFs should routinely be included in the capacity-building training sessions organised by the MoH and in workshops to disseminate health policies and national healthcare guidelines. Further research should be conducted on the effect of improving communication between LLPHFs and referral health facilities by affordable means, such as telephone, and the impact of community initiatives, such as transport vouchers, on promoting adherence to referral for sick children.

## Figures and Tables

**Table 1:** The health facility classification in Uganda.

Level of Health Unit	Target Population	Services Provided and Structures
Village health teams (Health centre I)	1000	First contact for populations living in rural areas, providing community-based preventive and health promotion services, community mobilisation and referral of sick members to health facilities. No physical structures.
Health centre II	5000	Parish-level facility offering disease prevention, health promotion and outpatient curative health services for uncomplicated conditions, antenatal care and immunisation for children.
Health centre III	20,000	Sub-county-level facility offering preventive, health promotion, outpatient curative, maternity, inpatient health services and laboratory services for malaria testing and tuberculosis microscopy.
Health centre IV	100,000	County-level facility offering disease preventive services, health promotion, outpatient curative, maternity, inpatient health services, emergency surgery and blood transfusion and laboratory services.
General hospital	500,000	District-level facility. In addition to services offered at HC IV, it offers general services and in-service training, consultation and research to community-based healthcare programs.
Regional referral hospital	2,000,000	In addition to services offered at the general hospital, it offers specialist services, such as psychiatry; ear, nose and throat; ophthalmology; dentistry; intensive care; radiology; pathology; and higher-level surgical and medical services.
National referral hospital	10,000,000	Offers comprehensive specialist services and is involved in teaching and research.

Source: Ministry of Health Annual Health Sector Performance report 2018–2019.

**Table 2:** Demographic characteristics of caretakers and healthcare providers.

Characteristic	Statistic
Caretaker participants (*N* = 16)	
Female sex	11
Average age	30.8 years
Relationship to child	
Mother	9
Father	5
Grandmother	2
Health worker participants (*N* = 30)	
Male sex	17
Female sex	13
Average period in practice in years	6.5
Nurses	18
Clinical officers	6
Doctors	3
Laboratory assistant	1
Nursing assistants	2

**Table 3:** Summary of the themes and subthemes for the referral process.

Theme	Subtheme	Codes
Reasons for referral	Severely ill child	Limited capacity to manage severe illness Limited knowledge and skills Limited investigative capacity Lack of oxygen and other treatments Lack of admission facilities
	Avoiding loss of revenue	Caretaker’s refusal to pay Caretaker’s lack of funds
	Loss of prestige	Fear to lose prestige if child dies at facility
Process of referral	Person responsible for referral	Assessing health worker Most senior health worker Proprietor of the health facility
	Where to refer	Regional referral hospital Specialised children’s hospital Physician in private health facility
	How are the referrals done	Referral notes Medical forms Verbally Physically taken by health facility staff
Health worker attitudes to referral	Positive	Results in good outcome for patients Increases trust from patients Provides opportunity to learn
	Negative feelings	Feeling incompetent Disappointing clients
Experiences of caretakers	Negative experiences	Incurring high costs for transport, medical care and feeding Difficulty in accessing transport Overcrowding on the ward spaces Unfriendly health facility staff Delays in accessing care
	Positive experiences	Possibility for child to receive appropriate care Caring health workers Free medical care
Challenges in referral	Non-adherence to referral instructions by caretakers	Refusal to go facility chosen by health worker Delay to take child to referral facility Complete refusal to take child to referral facility
	Loss of revenue to clinic	Failure or refusal of caretaker to pay for pre-referral care
	Lack of feedback from referral facilities	Lack of feedback concerning referrals from big hospitals
How the referral process could be improved	Reduce waiting time	Increase number of healthcare workers Give referral letters
	Improve transportation	Provide community ambulances Improve roads
	Reduce costs incurred	Establish referral health facilities nearer to communities
	Improved communication	Explaining to caretakers properly Improving communication between referring and referral health facilities

## Data Availability

The data that support the findings of this study are available from Juliet Mwanga-Amumpaire upon request.
